# Evaluation of the efficacy of a short-course, personalized self-management and intensive spa therapy intervention as active prevention of musculoskeletal disorders of the upper extremities (Muska): a research protocol for a randomized controlled trial

**DOI:** 10.1186/s12891-016-1353-8

**Published:** 2016-12-09

**Authors:** Charlotte Lanhers, Bruno Pereira, Chloé Gay, Christian Hérisson, Christine Levyckyj, Arnaud Dupeyron, Emmanuel Coudeyre

**Affiliations:** 1 Physical Medicine and Rehabilitation, University Hospital of Clermont-Ferrand (CHU), 58, rue de Montalembert, 63000 Clermont-Ferrand, France; 2 University of Clermont-Ferrand Auvergne, Auvergne University, 28, Place Henri-Dunant, 63000 Clermont-Ferrand, France; 3 Preventive and Occupational Medicine, University Hospital of Clermont-Ferrand (CHU), Clermont-Ferrand, France; 4 Clinical Research and Innovation Direction, University Hospital of Clermont-Ferrand (CHU), Clermont-Ferrand, France; 5 Physical Medicine and Rehabilitation, Hospital of Caremeau, University of Montpellier 1, 30029 Cedex 09 Nîmes, France; 6 Physical Medicine and Rehabilitation, University of Montpellier 1, Hopital of Lapeyronie, CHRU Montpellier, 371 Av. du Doyen Gaston Giraud, 34295 Cedex 5 Montpellier, France; 7 Research and Development, Thermal Cure Center de Royat, 1 place Allard, CS 20053 Royat, 63408 Chamalières Cedex, France; 8 INRA, Unity of Human Nutrition (UNH, UMR 1019), CRNH Auvergne, Clermont-Ferrand, France

**Keywords:** Musculoskeletal disorders, Exercise, Spa therapy, Prevention

## Abstract

**Background:**

Musculoskeletal disorders (MSDs) constitute a major occupational health problem in the working population, substantially impacting the quality of life of employees. They also cause considerable economic cost to the healthcare system, with, notably, the reimbursement of treatments and compensation for lost income. MSDs manifest as localized pain or functional difficulty in one or more anatomical areas, such as the cervical spine, shoulder, elbow, hand, and wrist. Although prevalence varies depending on the region considered and the method of assessment, a prevalence of 30% is found in different epidemiological studies. The disease needs to be prevented, not only for medical and economic reasons, but also for legal reasons, owing to the requirement of assessing occupational risks. The strategy envisaged may thus revolve around active, multimodal prevention that has employees fully involved at the heart of their care. Although physical exercise is widely recommended, few studies with a good level of evidence have enabled us to base a complete, well-constructed intervention on exercise that can be offered as secondary prevention in these disorders.

**Methods:**

A prospective, multicenter, comparative (intervention arm vs. control arm), randomized (immediate vs. later treatment) study using Zelen’s design. This study falls under active prevention of MSDs of the upper extremities (UE-MSDs). Participants are workers aged between 18 and 65 years with latent or symptomatic MSDS, with any type of job or workstation, with or without an history of sick leave. The primary aim is to show the superiority at 3 months of a combination of spa therapy, exercise, and self-management workshops for 6 days over usual care in the management of MSDs in terms of employee functional capacity in personal and professional daily life. Secondary aims are to assess the benefit of the intervention in terms of pain, quality of life, and accumulated duration of sick leave.

**Discussion:**

This randomized controlled trial is the first that will aim to evaluate multidisciplinary management of UE-MSDs using nonpharmacological treatment combining exercise, self-management, and spa therapy. The originality of this intervention lies, in its short, intensive format, which is compatible with remaining in work; and in its multidisciplinary approach. This trial has the potential to demonstrate, with a good level of evidence, the benefits of a short course of spa therapy combined with a personalized self-management program on the functional capacity, pain, and quality of life of employees in their daily life.

**Trial registration:**

Clinical trial.gov NCT02702466 retrospectively registered.

**Protocol**: Version 4 of 9/10/2015.

**Electronic supplementary material:**

The online version of this article (doi:10.1186/s12891-016-1353-8) contains supplementary material, which is available to authorized users.

## Background

Musculoskeletal disorders (MSDs) constitute a major occupational health problem in the working population, substantially impacting the quality of life of employees [1]. They also cause considerable economic cost to the healthcare system, [2]. MSDs pose difficulties for companies as well, including loss of productivity, accumulated sick leave, and employee absenteeism [3].

The term MSD currently lacks consensus. Both degenerative and inflammatory diseases can define this term [1]. MSDs manifest as either pain or functional difficulty in one or more anatomic areas [4]. Although prevalence varies depending on the regions considered and the methods of evaluation, a prevalence of 30% is found in different epidemiological studies [5]. MSDs of the upper extremities (UE-MSDs) represent two thirds of MSDs, because the upper limbs are at greater risk, and because of exposure to the offending economic sectors that are the supermarket sector, the food industry, and manufacturing companies [6]. Regardless of their location, MSDs may become chronic and lead to long-term disability. Risk factors may be external, involving repetitive biomechanical constraints [7] aggravated by stress, psychosocial factors, and work organization [8]. All these factors interact with each other and may contribute to worsening the pain or impairment. They are also intrinsic to the individual, including age and length of time in the position. Women are more often affected than men [9].

The disease needs to be prevented, not only, of course, for medical and economic reasons, but also for legal reasons, owing to the requirement of assessing occupational risks [10]. Primary prevention is mostly initiated by the company through ergonomic and organizational actions on occupational constraints, but it is far from sufficient. The actual impact of primary prevention continues to be debated in the literature [11].

The strategy envisaged may then revolve around active, multimodal secondary prevention that has employees fully involved at the heart of their care.

Performing exercises or a physical activity reduces MSD-related pain and impairment [12] while also limiting the increase in the risk of morbidity and mortality observed in this kind of population [13]. Although physical exercise is widely recommended, few studies with a good level of evidence allow us to base a complete, well-constructed intervention on exercise that can be offered as secondary prevention of these disorders [14].

Current recommendations for managing chronic pain highlight the benefits of multidisciplinary programs that combine various nonpharmacological treatments. These can potentiate each other to augment their benefits and prolong their positive effects over time [15].

Different studies have shown that self-management has a positive impact in the approach to MSD-related pain and incapacity [16]. The management of stress and pain through sophrology sessions is an additional asset in the therapeutic arsenal for this chronic disorder [17].

Spa therapies have long been used in the management of various rheumatological disorders like osteoarthritis, rheumatoid arthritis, or ankylosing spondylitis [18]. Another study has shown the benefit of balneotherapy on symptoms and quality of life in the management of fibromyalgia, notably by offering a combination of spa therapy, physical exercise, and self-management [19].

To our knowledge, the physical exercise–spa therapy combination has not been specifically studied in the area of UE-MSDs.

### Study objectives

This study falls under active UE-MSD prevention. Its primary aim is to show the superiority at 3 months of a combination of spa therapy, exercise, and self-management training for 6 days over usual care in the management of MSDs in terms of employee functional capacity in personal and professional daily life.

Secondary aims are to assess the benefit of the intervention in terms of pain, quality of life, and accumulated duration of sick leave.

This may lead to the possibility of proposing an alternative to conventional MSD treatment. The objective is to keep employees in work; the study design does not require sick leave. Decreased long-term use of analgesics is expected as well as a control of the unfavorable course of the MSDs. A resurgence in pain and fatigue immediately after the week of treatment is expected to be a possibility.

## Methods

### Subject selection

**Fig. 1:**
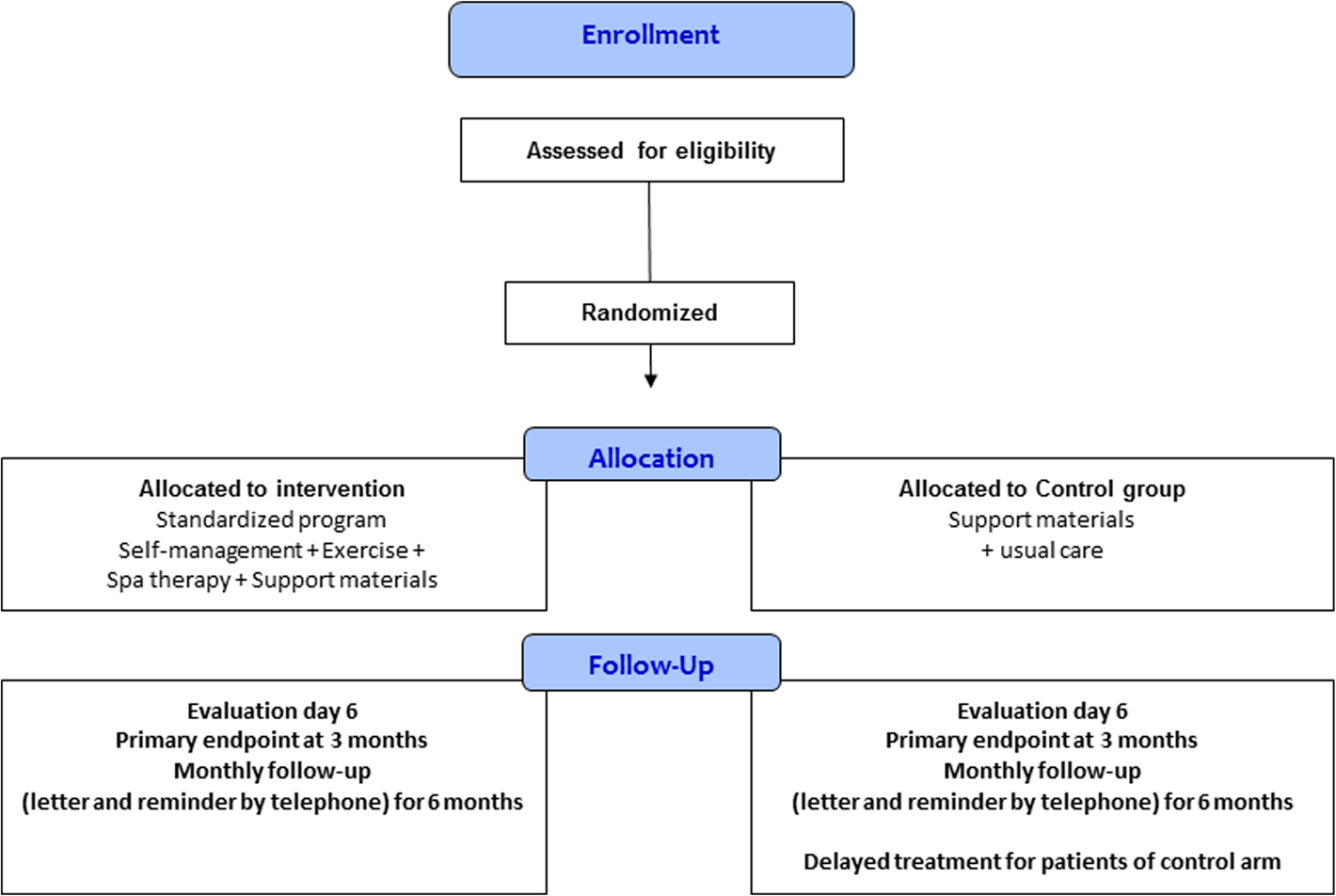
Flow chart

Recruitment is preferably by posters, media and occupational health services. Following a double assessment (knowledge of the occupation and health status of employees), occupational physicians are the best placed to identify and recruit employees potentially eligible for the study. A more conventional method of recruiting during consultation will also be conducted by general practitioners who have agreed to participate in the study (Fig. 1). Recruiting locally is a necessity because the study targets working people. Thus, patients may carry out the treatment before or after work and return home daily.

### Selection criteria

#### Inclusion criteria

Participants are workers with latent or symptomatic MSDs according to the SALTSA European consensus/INRS (French occupational health and safety agency) [20] with a value on the Nordic questionnaire strictly above 2/10 [21]. They are aged between 18 and 65 years, have or have not a history of sick leave, and are covered under national health insurance. No restriction was made regarding type of job or workstation.

#### Exclusion criteria

Workers who are contraindicated to spa therapy or who underwent spa therapy within the last 12 months are not included. Nor are people with behavioral disorders or comprehension difficulties that make assessment impossible (Table 1).

**Table 1:** MUSKA study eligibility criteria

Inclusion criteria
-Latent or symptomatic UE-MSD according to the SALTSA European consensus/INRS (French occupational health and safety agency) with a score on the Nordic questionnaire >2
-With or without a history of sick leave and working at the time of inclusion
-Age: >18 years <65 years
-Patient covered under national health insurance (person insured or dependent)
-Patient has given informed written consent to participate in the study
Non inclusion criteria
-Contraindication to spa therapy:
Unstable angina
Myocardial infarction within the previous 6 months
Cerebrovascular accident within the previous 6 months
Recent thrombophlebitis
Attack of inflammatory rheumatism
Progressive cancer
Vascular surgery within the previous 6 months
-Course of spa therapy within the previous 12 months
-Behavioral disorders or comprehension difficulties making assessment impossible

The research may be discontinued early if a contraindication to spa therapy or issues relating to participant availability arise. No exclusion period is planned in this study. No adverse effect is expected. Before each spa therapy session, the contraindications described in the non-inclusion criteria will be checked.

Travel expenses arising from participation in the study will be fully reimbursed at a fixed rate.

### Instruments

The primary endpoint is the score on the Quick DASH scale at 3 months. The Quick DASH (Disability of Arm-Shoulder-Hand) is a self-administered questionnaire which measures the physical disability and symptoms for all upper limb disorders in an heterogeneous population and for acute as well as chronic disorders [22]. It contains various domains such as handicap, activity of daily living, pain during activities, strength. It has good psychometric properties with a Cronbach’s alpha coefficient of 0.89 and an intra-class correlation coefficient of 0.94 which suggests excellent test-retest reliability. We assessed functional capacity monthly over 6 months.

Secondary endpoints are:

- The areas of pain and intensity of the discomfort or pain are assessed using the Nordic questionnaire [21] and a numerical pain scale.
- Overall psychological status is assessed using the Hospital Anxiety and Depression Scale by Zigmond and Snaith [23].
- The SF-36 questionnaire is used for patient quality of life [24].
- Duration of sick leave accumulated during the study period.
- Use of analgesics.

### Research design

A prospective, multicenter, comparative (one intervention arm vs. one control arm), randomized (immediate vs. later treatment) study using Zelen’s design [25]. Workers randomized to the intervention group will be given a short, intensive, standardized spa therapy treatment during the following month. The intervention will be offered to control-group subjects at least 3 months later. The total duration of participation in the study for patients is 6 months. The centers were Clermont-Ferrand University Hospital/Royat spa center, Nîmes and Montpellier Hospital/Balaruc spa center.

### Description of measures taken to avoid bias

Given the context, blinding workers, physicians, and assessors is impossible. To control bias arising from the absence of blinding, we propose using a modified version of Zelen’s design. This makes it possible to prevent the risks of bias caused by the absence of blinding, because in conventional randomized trials workers assigned to the control group may withdraw from the trial more easily, try to receive the study treatment, or change their behavior when the endpoint is being assessed. The primary endpoint was blind evaluated.

The risk of contamination in the usual-care group is highly unlikely because our short spa therapy program is not currently offered outside of this study.

The risk caused by revealing the study hypotheses to workers is reduced by restricting contact between the patients of the two groups.

### Randomization

The randomization list will be drawn up by the methodologist in charge of the project before the beginning of the trial. Randomization will be conducted so as to balance group numbers using block randomization stratified by center; this will make it possible to check patient eligibility and to communicate information on randomization to the investigator and any other correspondents for each of the workers.

### Sample size determination

To study the impact at 3 months of the 6-day standardized short thermal intervention on pain-related functional impairment measured using QUICK DASH scale (disability/symptom section), the sample size estimation is based on a comparison between both arms for a two-sided type-I error of 5 and 90% statistical power. According to the literature [26], it seems relevant to consider a standard deviation at 18 and a baseline value equaling 48 (/100) for the QUICK DASH scale. Considering (i) an assumed 25% improvement (−12 points) in favor of the 6-day standardized short thermal group, (ii) a possible Hawthorne effect (in the “delayed treatment” group), and (iii) a lost-to-follow-up rate of 20%, it is planned to include 75 patients per randomization group. Data will be entered in real time using the online clinical research data management tool REDcap (Research Electronic Data Capture). Assessment questionnaires will be data-scanned to prevent data entry risks.

#### Procedure for data collection

The study inclusion visit will enable the collection of sociodemographic parameters (age, weight, height) and job characteristics (profession, name of company, length of time in the position, stress, and interest in the position).

#### Conduct of the study

**Fig. 2:**
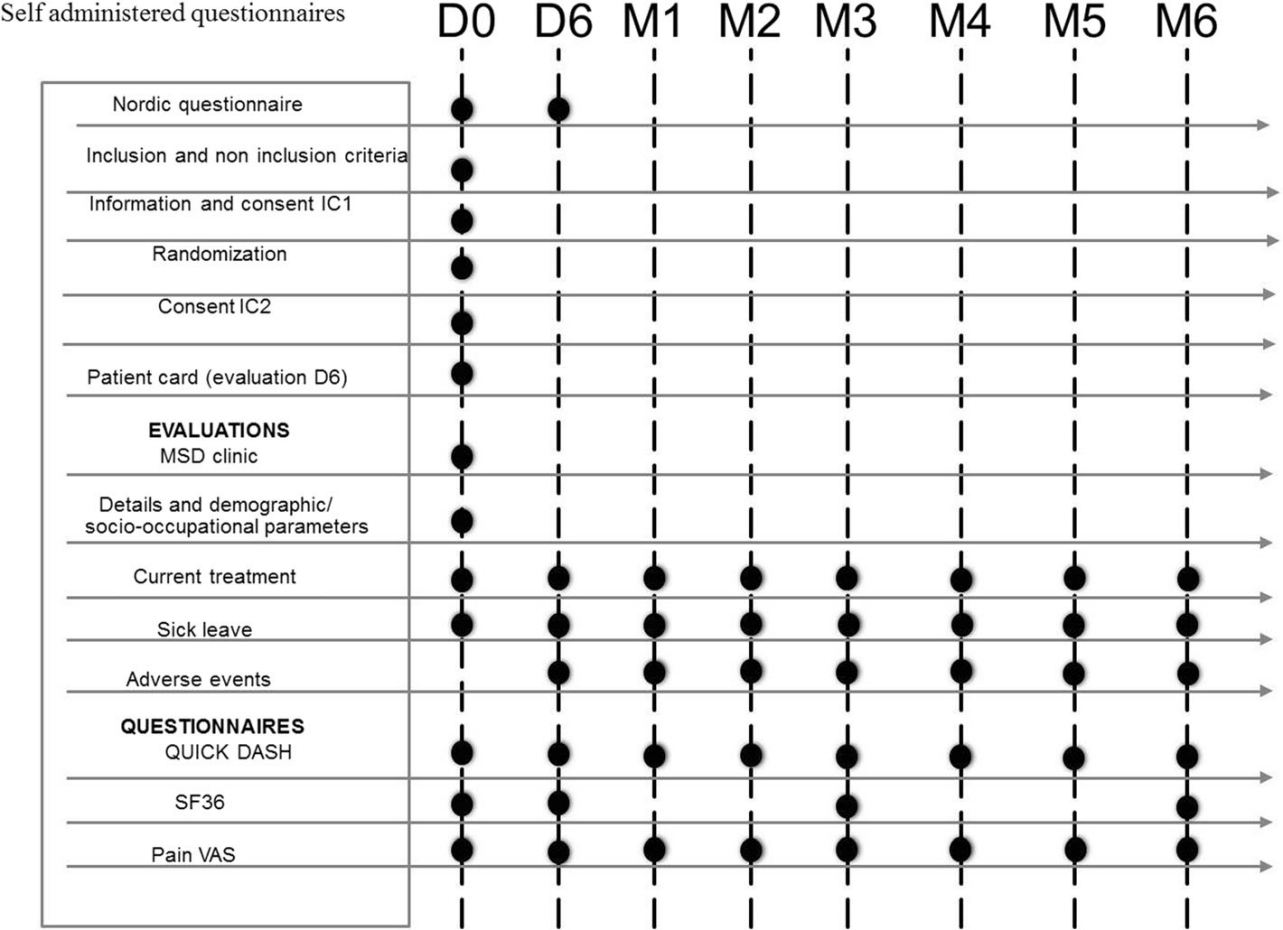
Self administered questionnaires

The self-administered questionnaires are repeated during the study at predetermined intervals over a period of 6 months (Fig. 2).

Quantitative adherence (number and duration of sessions) and qualitative adherence (type and way of performing the intervention) will be systematically evaluated using monthly follow-up self-administered questionnaires [27, 28].

The assessment of endpoints on day 6 and then monthly over 6 months will be conducted by sending self-administered questionnaires by mail. Reminders and follow-up will be by mail and by telephone calls if necessary.

### Ethical consideration

All workers will receive their written consent form, which they will sign all with the physician during the inclusion medical visit. The study will be conducted in accordance with the principles of the Helsinki declaration. This protocol was approved by the Sud-Est VI medical ethics committee of the University Hospital of Clermont-Ferrand, France, institutional review board (reference: AU 1206 – IRB no: IRB00008526) on July 3, 2015, as well as by the French National Agency for Medicines and Health Products Safety on August 14, 2015.

### Intervention protocol

The spa therapy and individualized self-management interventions have been put together by a steering committee and then standardized so as to be the same in the different centers participating in the study. The steering committee, which comprises investigators, treatment managers, physiotherapists, sports instructors, and psychologists, was in charge of the standardization and reproducibility of the intervention for all the centers.

**Fig. 3:**
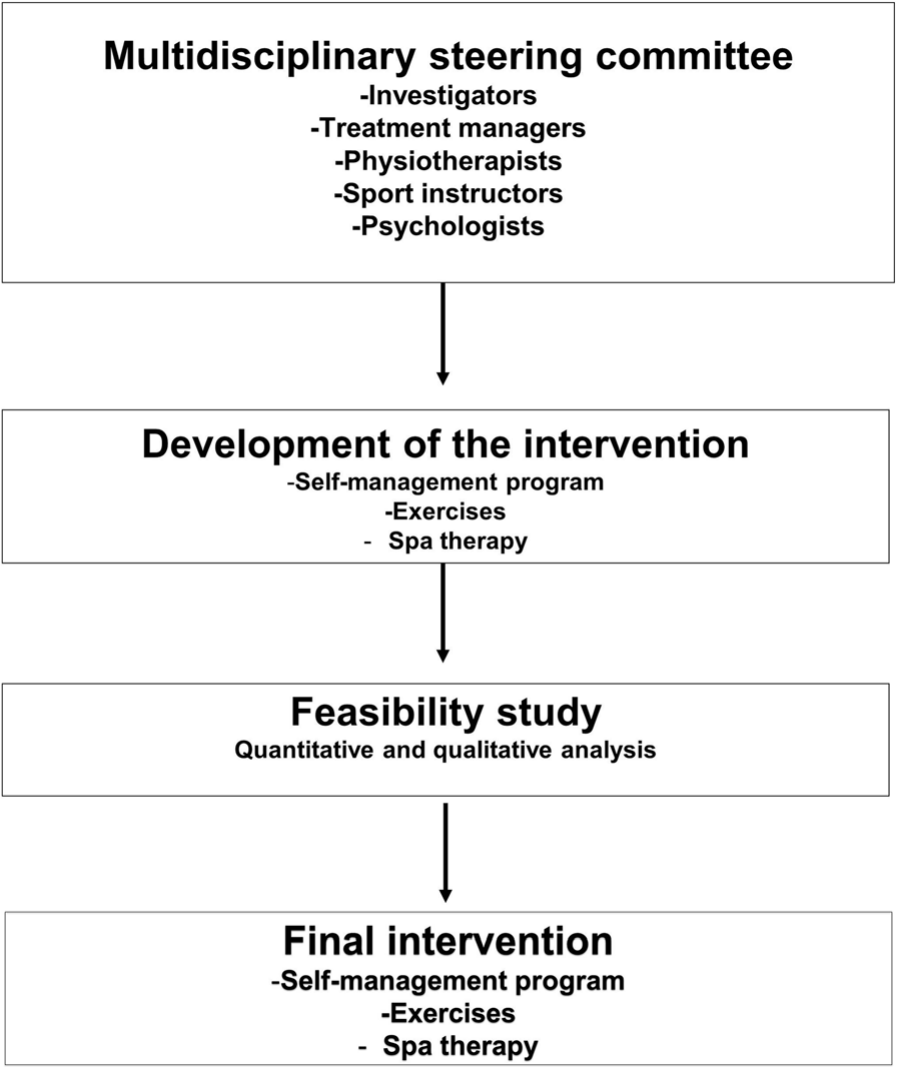
Intervention

Meetings with all the steering committee members were organized to find a consensus on the treatment protocol, which describes in detail the content and organization of the intervention while keeping in mind the circumstances and expertise of the different centers involved in the study. This content was approved by means of a feasibility study before the study began. The content of the intervention is defined both in the written protocols, which are illustrated with photographs, and in digital format as a USB key, with recordings from the feasibility assessment week (Fig. 3, Additional file 1: Figure S1, Additional file 2: Video S1).
**Additional file 2: Video S1.** Cervical spine stretch video. (MP4 46804 kb)

Intervention-group workers will receive the spa therapy at the beginning of the study. After the spa therapy, workers will continue their home exercise program up to the end of the study.

Concerning the spa therapy, they will receive 2 h of intensive therapy for 6 consecutive days: 1 h of spa therapy, a break of 15 min, and a 45-min self-management session. The spa therapy will be outside the working hours. Study workers were not mixed with the general public in the spa center.

Spa mineral water and treatments are approved and controlled by the French authorities (Table 2). Treatment included: mineral hydrojet sessions at 37 °C for 10 min, manual massages of upper-limbs under mineral water at 38 °C for 10 min, applications of mineral matured mud at 45 °C to the upper-limbs for 15 min and supervised general mobilisation in a collective mineral water pool at 32 °C in groups of seven patients for 15 min.

**Table 2:** Spa therapy treatments

Description	Duration	Conducted by
Underwater massage bath	10 min	Spa therapist
Underwater jet massage with focused massaging of the area of pain	10 min	Spa therapist
Mud wraps or similar (poultice)	15 min	Spa therapist
Hydrotherapy session	15 min	Spa physiotherapist

The treatments are prescribed during the study inclusion visit and are tailored based on the areas of pain identified during clinical examination. They are performed by spa therapists and physiotherapists.

The self-management program consists of sessions of physical exercises out of the water without any special equipment. It is run by a physiotherapist or adapted physical activities instructor on days 1, 3, and 5. The physical activity sessions alternate with counseling with a psychologist on pain management and relaxation on days 2, 4, and 6.

Workers also receive a folder containing sheets with physical exercises adapted to their condition so that they may continue a stretch break program after the course of treatment, relaxation exercises that they can perform themselves, as well as a full program of health activities on a USB key. The sheets in this folder are illustrated with photographs and detailed descriptions of the exercises to perform. The most relevant exercises for each worker will have been individually determined during the week of treatment.

The various people in charge of the spa therapy who are responsible for conducting the intervention in this study, have more than 3 years of clinical experience and have been specifically trained in implementing the protocols by means of a detailed handbook that precisely describes each intervention illustrated with photographs and diagrams from the video program given to workers [27].

During the inclusion visit, control-group workers will be given the same folder as the intervention group, with the seven exercise sheets and USB key. The workers are not given guidance on the performance of any exercise that may have been determined by the conduct of the clinical examination. They will perform home program (exercises and relaxation) during the first three months and then the spa therapy of 6 consecutive days identical to the intervention group.

Intervention and control-groups workers will receive unrestricted pharmacological or non-pharmacological usual care from their physicians or specialists, such as analgesics, physiotherapy or orthosis, over 6 months. However, any medication taken during or after the intervention must be declared and reported in the patient follow-up questionnaires.

#### Statistical analysis

Statistical analysis will be conducted on the intention-to-treat population using Stata software, V.13 (StataCorp, College Station, Texas, USA). A two-sided P value of less than 0.05 will be considered for statistical significance. Baseline characteristics will be presented for each randomized group as the mean ± standard deviation or median (interquartile range) according to the statistical distribution for continuous data, and as the number of patients and associated percentages for categorical parameters. Comparisons between randomized groups will be analyzed using the Chi2 or Fisher’s exact test for categorical variables and the Student t-test or Mann-Whitney’s test for quantitative parameters, with normality verified by the Shapiro-Wilk test and homoscedasticity by the Fisher-Snedecor test (notably for primary outcome QUICK DASH scale at 3 months). Primary analysis will be completed using (1) ancova taking into account baseline QUICK DASH as covariate as recommended by Vickers and Altman [29] and (2) linear regression model in multivariate context. Covariates used for adjustment will be fixed according to (i) univariate results and (ii) clinical relevance including (use of analgesics) stratification factors. Results will be expressed as effect sizes and 95% confidence intervals. Regarding the analysis of repeated measures, random-effect models (linear or generalized linear according to the statistical distribution of dependent variables) will be performed, as usually proposed, to study the fixed-effects group, time points, and “group x time” interaction, taking into account between- and within-subject variability. The impact of covariates (as observance) will be also studied. A sensitivity analysis will be performed to study the impact of missing data; when appropriate, they will be handled in accordance with the estimation method developed by Verbeke and Molenberghs [30].

## Discussion

This randomized controlled trial is the first that will aim to evaluate multidisciplinary management of UE-MSDs using nonpharmacological treatment combining exercise, self-management, and spa therapy. The conduct of this study is important due to the absence of consensus or guidelines for the management of UE-MSDs and its medical and economic major issue. Moreover, despite numerous small scale studies, high quality scientific evidence for the efficacy of spa therapy for MSDs is lacking.

The mechanisms of action behind spa therapies in the treatment of pain are complex and as yet have only been partially elucidated. In workers with osteoarticular diseases, poultices and immersion in water have been shown to improve functional capacities and quality of life, and to lower the intensity of pain perceived [31–35].

Spa therapies induce mental relaxation, reduce anxiety, and so procure a feeling of well-being, which is an important factor in the prognosis of pain progression and chronic disorders because it improves quality of life [36, 37].

Exercising or being physically active must be the key element in chronic pain treatment, according to current recommendations [38]. Yet many obstacles to taking part in physical activities have been described in the literature, such as the fear of injury, pain, or misconceptions [39, 40] in this kind of population. The chronic nature of MSDs results in distorted thought processes often summarized by the general term catastrophism. Hence it is difficult to change the behavior of employees on partaking in physical activities. Workers thus need to be guided through a tailored, personalized care strategy, while adherence to treatment needs to be optimized. Information and education based on the biopsychosocial model are effective strategies for changing beliefs, minimizing repercussions, and increasing adherence to treatment. Thus, psychological counseling and self-management programs (which often happen in groups, for instance during spinal functional restoration programs in chronic lower back pain [41]) are primordial for developing adaptation strategies to reduce functional incapacity even if the pain is still present.

MSDs are one of the main causes of long-term sick leave, representing 34 and 17%, respectively, of sick leave in manual and nonmanual workers [42]. These periods of sick leave are a major economic cost to society [3].

Having said that, the various treatments undertaken outside and within the work environment to keep workers at work or facilitate their return to work have their cost too [43]. A recent study evaluated the cost-effectiveness of three types of treatment for MSD aiming to facilitate the return to work of employees on long-term sick leave [42]. The interventions consisted of a physical activity and education intervention, a workstation intervention, or a combination of physical activity, education, and workstation assessment. This modeling approach showed that the most cost-effective intervention was physical activity and education. Given the current importance of the economic impact of chronic disorders and their treatment, our study will include a supplementary medical cost-effectiveness analysis with a societal perspective to evaluate whether it will be possible to implement our global multidisciplinary intervention [44]. Cost estimation will include direct and indirect medical costs. The cost of spa therapy will be calculated from guidelines of the French association of thermal centers. An economic analysis society’s willingness to pay for the thermal water treatment would be also measured.

The originality of this intervention lies, firstly, in its short, intensive format, which is compatible with remaining in work; secondly, in its multidisciplinary approach; and lastly, in its target population. The program is aimed at a population who are in employment at the time of treatment, so patients do not need to be on sick leave to follow treatment. This is made possible by the duration of the intervention (1 week), by the proximity of the centers to the workplace or residence of patients, and by the flexibility of treatment times relative to patients’ work schedules.

Inclusion of subjects commenced in September 2015 and will continue until February 2018. The study is projected to end in September 2018. Results should be available in February 2019.

This trial has the potential to demonstrate, with a good level of evidence, the benefit of combining exercise, a self-management program, and spa therapy in the therapeutic arsenal of UE-MSDs. In accordance with the literature, concerning exercise and education, we hope to show the benefits of a short course of spa therapy combined with a personalized self-management program on the functional capacity, pain, and quality of life of employees in their daily life. Standardizing the intervention will make it possible to develop and generalize a precise management plan that can be offered early to employees who present with these disorders when they consult a secondary care physician. This possible treatment may also be an alternative to keeping employees at work for occupational physicians forced to contend with the incapacities of their employees in the world of work [45].

## Additional files

Additional file 1:
**Figure S1.** Education sheet neck. (PDF 786 kb)

## Abbreviations

MSD: Musculoskeletal disorder
UE-MSD: Musculoskeletal disorder of the upper extremities
