# Supplementary material for: Evaluation of the efficacy of a short-course, personalized self-management and intensive spa therapy intervention as active prevention of musculoskeletal disorders of the upper extremities (Muska): a research protocol for a randomized controlled trial
Source: BMC Musculoskelet Disord. 2016 Dec 9;17:497. doi: 10.1186/s12891-016-1353-8 (PMC5148841; doi:10.1186/s12891-016-1353-8)
Supplement: Additional file 1: — Figure S1. Education sheet neck. (PDF 786 kb) [file 12891_2016_1353_MOESM1_ESM.pdf]

S1Fig. Education sheet neck

MUSKA TMS - MS

EDUCATION SHEET NECK

TIPS

- Exercise at your **own pace**, **on a regular basis** and in accordance with your **goals**.
- You can adjust the **duration**, **rest periods** and **number of repetitions.**

Duration of exercises 2 min

Why the neck?

Neck muscles contract not only to keep the head aligned with the spine but also to compensate for the eye strain forcing you to bring your head forward.

Why carry out these exercises?

- Relax neck muscles and avoid torticollis
- Maintain mobility

1 Neck massage

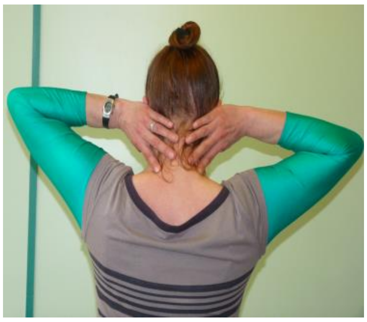

- Place your fingers on each side of the skull
- Move your fingers from the base of the skull down along the neck
- Massage in circles

2 Cervical spine stretch

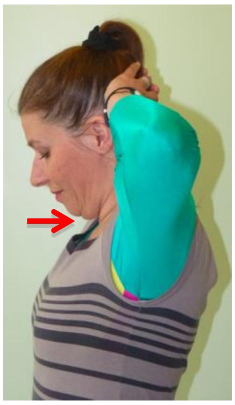

- Place your hands on the back of your head
- While exhaling, tuck your chin into your chest

3 Head rotation

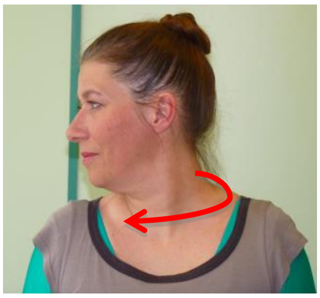

- While exhaling, turn the head slowly to the right
- Return to the center
- While exhaling, turn the head slowly to the left

4 Neck mobility

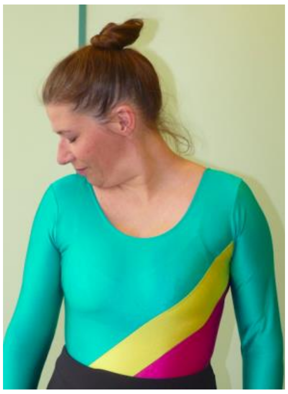

- Tilt the head downward
- Move the head in semi-circles

Keep your back straight

Duration **15-25s** *Number of repetitions* **10** *Pause and hold* **5-10s**

Symptoms

Which exercises?

Symptom	Exercises	Symptom	Exercises
Neck pain	2, 3	*Neck stiffness*	1, 4
Torticollis	1, 2, 4	*Headache*	1
Eye strain	2, 4	*Shoulder pain*	3
